# Surveillance Protocols After Nephrectomy or Ablation in Renal Cancer: A Scoping Review of Variations, Evidence, and Research Gaps

**DOI:** 10.7759/cureus.97554

**Published:** 2025-11-23

**Authors:** Mayowa Adefehinti, Onyiyechi Roseline Agwu, Kelvin Tetteh Ahulu, Adeogo B Adedeji, Steve Ndonga, Joshua Makinde, Adewale Jayeola, Saleh Nedjim

**Affiliations:** 1 Urology, Peterborough City Hospital, Peterborough, GBR; 2 Surgery, Lagos University Teaching Hospital, Lagos, NGA; 3 General Practice, Rhema Rapha Medical Centre, Accra, GHA; 4 General Practice, Northern General Hospital, Sheffield, GBR; 5 Surgery, Airedale General Hospital, Steeton, GBR; 6 General and Colorectal Surgery, Norfolk and Norwich University Hospitals NHS Foundation Trust, Norwich, GBR; 7 Urology, Centre Hospitalier Universitaire (CHU) Pointe à Pitre, Pointe à Pitre, GLP

**Keywords:** evidence-based medicine (ebm), imaging and diagnostics, postoperative surveillance, renal cell carcinoma, urological oncology

## Abstract

Guidelines and surveillance practices after nephrectomy or renal cell carcinoma (RCC) ablation are very diverse across institutions. It has been proven that close follow-up is essential to identify local recurrence, metastatic spread, and treatment-related complications, but the frequency, length, and imaging modalities employed in surveillance are widely different in terms of practices.

Although these guidelines have been advised by larger organizations like the American Urological Association (AUA) and the European Society for Medical Oncology (ESMO), they are mostly risk-stratified and lack comparative data. Entirely new imaging methods (such as multiparametric MRI and state-of-the-art contrast-enhanced CT) have been proposed to enhance early disease diagnosis and are currently under consideration regarding their cost-effectiveness and long-term results. Simultaneously, some minimally invasive ablative forms of treatment, such as radiofrequency and cryoablation, have complicated surveillance measures, given that recurrence patterns differ from those seen after conventional nephrectomy.

Although systematic reviews have found the oncological safety of active surveillance in specific small kidney masses, research still shows loopholes regarding regular follow-up and outcome reporting that is patient-centered. Out of 170 records initially identified, 140 unique articles were screened after duplicate removal, with 30 assessed in full text. Following exclusions, five studies were finally included in the review. These comprised a mixture of clinical guidelines, systematic reviews, and observational studies, highlighting the heterogeneity of follow-up schedules, imaging-based approaches, and patient risk stratification.

This scoping review maps the existing literature on surveillance following nephrectomy or ablation in RCC. The review establishes differences in practice, evidence, and research gaps that need to be harmonized to enhance long-term management.

## Introduction and background

Renal cell carcinoma (RCC) is the most common type of kidney cancer and remains a significant global health concern, accounting for approximately 2-3% of all malignancies worldwide [1]. Its incidence has been gradually increasing, in part due to the widespread use of cross-sectional imaging, which frequently detects small, asymptomatic renal masses incidentally. This shift in diagnostic patterns has influenced management strategies, as more localized tumors are now being identified at earlier stages [2].

Surgical resection of localized RCC has historically been an effective treatment method. It constitutes the gold standard since it offers long-term control of these cancerous cells, and it is probably a curative option [3]. However, less invasive ablative radiofrequency ablation (RFA) or cryoablation has also surfaced and been refined within the past 20 years, and is also a possibility for those patients with comorbidities or nonoperative causes [4]. This diversification of the treatment routes has inevitably caused new questions about how it is best to monitor patients in the postoperative/post-ablation setting, especially given certain peculiarities of recurrence with each modality [5].

An additional purpose of surveillance following nephrectomy or ablation is not only to identify local recurrence at an early stage, and the presence of metastasis, but also functions as a means to assess functionality. Meanwhile, follow-up procedures are planned to minimize the exposure to radiation in addition to costs and challenges among patients that may include frequent movement. This must be taken into consideration when developing new policies, guidelines, or protocols that can be resource-efficient and lead to significant clinical outcomes [6].

Although there is consensus with respect to the necessity of surveillance, there is also significant diversity with respect to practice. One case in point is the recommendations behind the implementation of the American Urological Association (AUA) approach, which proposes a risk-based strategy, such as increased-risk patients getting more intensive schedules, and low-risk patients having more relaxed ones [3]. In fact, the European Society for Medical Oncology (ESMO) tries to concentrate on routine follow-up care and the use of state-of-the-art imaging in the detection of recurrence at the first viable location [7]. The existence of conflicting guidance represents the lack of strong supporting evidence, and it becomes the responsibility of clinicians to tailor surveillance activities to local experience, patient preference, and institutional capacity [8].

Imaging modality is another important aspect that deserves consideration. Contrast-enhanced CT has long been regarded as the foundation of surveillance due to its high sensitivity in detecting recurrence and metastasis [9]. However, adverse effects, such as cumulative radiation exposure and contrast-induced nephropathy, are increasingly concerning, particularly in patients with impaired kidney function after surgery. To address these issues, newer modalities, including diffusion-weighted MRI and multiparametric imaging protocols, are under investigation. These approaches have the potential to reduce harm without compromising diagnostic quality [10]. Nonetheless, their adoption remains inconsistent, as some academic centers have incorporated them into routine surveillance while many community hospitals have not.

When compared to surgery, treatment in terms of surveillance is also very different following ablation. There are recurrence rates in both of them following either RFA or cryoablation, which are often in the mild form of residual tumor at the ablation margin as compared to the fully comprehended local recurrence following the nephrectomy procedure [5]. To do this, it will involve protection with greater rates of early imaging to detect full ablation, and precision longitudinal follow-up. They underpin such tailor-made interventions as the professional societies, such as the Society of Interventional Radiology (SIR), notice that there is a dearth of information on such interventions in terms of quality [11]. This dependence on institutional procedures or physician bias also helps support the inconsistency in practice presently.

Surveillance is also affected by patient-centered issues. According to research, reassurances based on continual follow-up are appreciated by many individuals, and anxiety regarding waiting for imaging results is also reported [12]. Another area that still has gaps that need to be addressed in future research is balancing psychological outcomes and clinical necessity in the literature. Also, oncological outcomes are the focus of the surveillance discourse; however, functional outcomes, including the retention of renal function and long-term cardiovascular health, matter and need to be monitored in a systematic manner [8].

In RCC detection, the monitoring of the patient is understood to be vital, but the existing practice is dispersed and quite inconsistent in guidelines, facilities, and patient categories. The imaging and ablative technology has been out of touch with strong enough, evidence-based follow-up measures, either because there remains a lot of speculation among clinicians and patients [13]. This scoping review will discuss and summarize available information regarding the current surveillance practices and identify the practical and research gaps that need to be bridged to achieve the best possible clinical outcomes.

## Review

Review design

The review took a scoping design to undertake a systematic mapping of the literature on the topic of surveillance protocols following nephrectomy or ablation in renal cancer. Scoping reviews are especially practical in the context of domains, where the evidence base is heterogeneous, where guidelines are dissimilar, and gaps in the research can be observed [1]. The intention was not only to synthesize the familiar knowledge but also to discover variations and gaps that still remain sources of concern in practice.

Protocol and registration

The review was done in accordance with the framework suggested by Arksey and O’Malley, with the assistance of guidelines developed by Preferred Reporting Items for Systematic Reviews and Meta-Analyses (PRISMA) Extension of Scoping Reviews (PRISMA-ScR) [14]. The protocol was not officially registered in the International Prospective Register of Systematic Reviews (PROSPERO); therefore, it was designed in a way that would ensure transparency and reproducibility [2].

Eligibility criteria

The inclusion criteria were peer-reviewed articles, clinical trials, systematic reviews, and practice guidelines (2017 to 2025). Only studies written in English and focusing on surveillance after nephrectomy, partial nephrectomy, or minimally invasive ablation (radiofrequency, cryoablation, microwave) for renal cell carcinoma were included [4]. Studies exclusively examining systemic therapy or metastatic disease management were excluded.

Information sources and search strategy

The search strategy involved Google Scholar, PubMed, and Scopus to capture a wide range of peer-reviewed studies and guidelines. Search terms combined Boolean operators and Medical Subject Headings (MeSH), including “renal cell carcinoma,” “nephrectomy,” “ablation,” “surveillance,” “follow-up,” “protocol,” and “recurrence” [5]. Reference lists of included articles were screened to identify additional relevant publications.

Study selection

**Figure 1:**
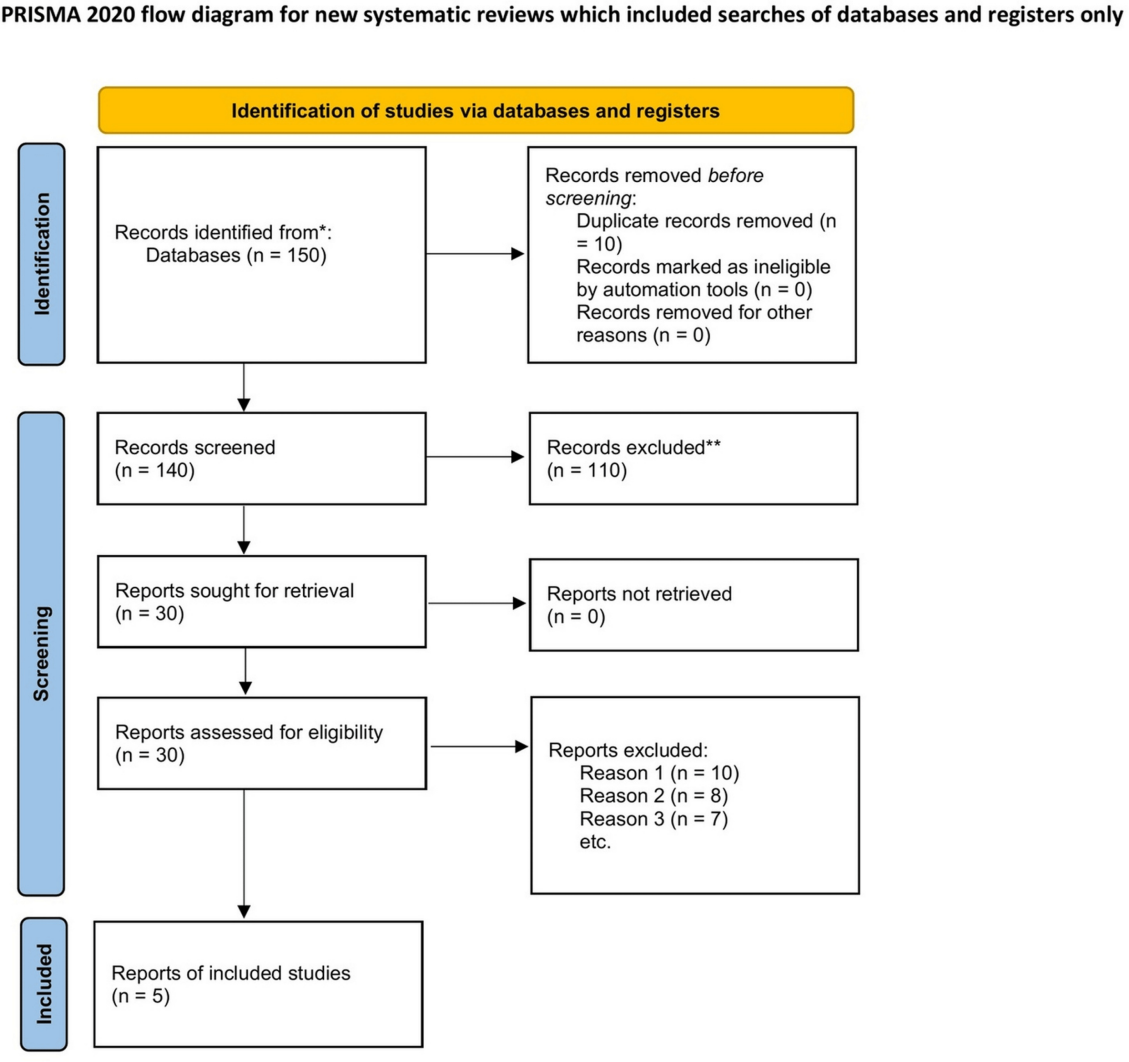
PRISMA flowchart. PRISMA: Preferred Reporting Items for Systematic Reviews and Meta-Analyses.

Articles were screened in two stages: title/abstract screening, followed by full-text review. Duplicates were removed, and two reviewers independently assessed eligibility. Discrepancies were resolved through discussion and consensus, ensuring that only studies directly relevant to postoperative or post-ablation surveillance were retained [15]. This is represented in the PRISMA flow chart in Figure 1 below.

Data charting process

Data were charted using a standardized template. Extracted variables included study design, year, setting, sample size, treatment modality, surveillance protocol used, imaging modality, follow-up duration, recurrence outcomes, and key findings [9]. Charting was conducted independently by two reviewers and cross-checked for accuracy.

Study characteristics

The included studies represented a blend of guidelines, systematic reviews, cohort studies, and narrative reviews. Most originated from North America and Europe, reflecting where surveillance guidelines are most developed. Sample sizes range widely, from single-center series of fewer than 100 patients to multi-institutional datasets involving thousands [3].

Table 1 below presents study designs, patient populations, treatment types, and key findings, highlighting significant variation in imaging schedules and follow-up intensity across surgical and ablative approaches.

**Table 1:** Study characteristics. RCC: renal cell carcinoma; RFA: radiofrequency ablation; SRM: small renal mass; AUA: American Urological Association; MRI: magnetic resonance imaging; AS: active surveillance.

Author (year)	Design/type	Population	Treatment type	Surveillance protocol	Key findings
Lam et al. (2020) [5]	Retrospective cohort	152 patients	RFA for SRM	CT at 3, 6, and 12 months, then annually	Recurrence mainly at ablation margins; early intensive imaging is critical
Filippiadis et al. (2019) [4]	Narrative review	Mixed	Cryoablation & RFA	Heterogeneous protocols	Ablation requires tailored, modality-specific surveillance
Campbell et al. (2021) [3]	Clinical guideline (AUA)	Localized RCC	Nephrectomy/partial	Risk-stratified follow-up with CT or MRI	Structured surveillance reduces late relapse risk
Bellin et al. (2024) [10]	Imaging review	RCC patients	All modalities	Advanced MRI protocols	Novel imaging offers early detection, but limited outcome data
Tsuboi et al. (2025) [13]	Systematic review	3,000+ patients	Surgery, ablation, AS	Protocols highly variable	Active surveillance can be safe, but lacks standardized schedules

Overview of included studies

Out of 170 records initially identified, 140 unique articles were screened after duplicate removal, with 30 assessed in full text. Following exclusion of 25 for reasons such as non-relevance to surveillance or focus on metastatic disease, five studies were finally included in the review [5]. These comprised a mixture of clinical guidelines, systematic reviews, and observational studies, reflecting the breadth but also the limitations of available evidence. In combination, the research studies indicated the heterogeneity in the follow-up schedules, imaging, and patient risk stratification [4].

Delays in presentation and diagnosis

Retrospective cohort research findings showed that recurrence in ablation is usually present in the form of residual disease in the first year, with the need to conduct early imaging [5]. Recurrence in cases undergoing nephrectomy, on the other hand, can occur many years following the operation; hence, extreme care observing any alterations will be required in the long term. The absence of a standardized schedule implies that a few categories of people might not receive a timely diagnosis, especially those who have not had an imaging schedule that often [2].

Predictive factors influencing recurrence detection

Risk stratification was another theme that emerged in all guidelines. According to the AUA guideline recommendations, more vigilant monitoring was suggested in high tumor grade or stage patients; according to the ESMO recommendation, it was proposed to monitor all patients within the first two years [1]. Proposed lengthy-primer diffusion-weighted MRI, along with other new technologies, became promising tools that are expected to diagnose early recurrence, but the data remains scarce [10].

Diagnostic challenges and negative investigations

The poor follow-up is also a barrier to diagnostic uncertainty. It can be difficult to tell where the tissue is scarring and where it is a viable tumor, and in any case, that may also result in further imaging or even recurring biopsies, when ablation patients are concerned [11]. Similarly, in the same spirit, the incidental nodules that are often found in the lungs require follow-up, and the uncertainty regarding their clinical significance, in turn, often prompts anxiety in the patient [6].

Recurrence and management outcomes

The recurrence rate was slightly higher after ablation in the studies mentioned, but in both methods, good long-term survival was observed, provided correct surveillance was used [13]. Table 2 below outlines the different outcomes between nephrectomy and ablation as far as surveillance is concerned.

**Table 2:** Comparative outcomes of nephrectomy vs. ablation surveillance.

Outcome	Nephrectomy (n = 3 studies)	Ablation (n = 2 studies)	Notes
Typical recurrence window	2–5 years post-surgery	Within 1–2 years post-ablation	Early intensive imaging critical [2,5]
Imaging modality of choice	CT or MRI annually	CT/MRI at 3, 6, and 12 months, then yearly	Margin recurrences are common [3,10]
Reported recurrence rates	3–7%	6–12%	Higher after radiofrequency ablation than cryoablation [4,13]
Salvage interventions	Completion nephrectomy	Repeat ablation or surgery	Both show preserved overall survival [5,13]
Patient anxiety/psychological load	Moderate	Higher due to uncertainty	Often linked to imaging frequency [2,6]

Trends

In general, there is a tendency in favor of more personalized surveillance pathways based on risk stratification, tumor biology, and patient comorbidities [15]. Although standard CT-based schedules are the main foundation of follow-up, MRI and ultrasound are increasingly popular with a view to reducing radiation load, especially among young patients. More so, better focus is currently on the functional results, like saving renal function and reporting on quality of life, which is not relatively well-researched in comparison with oncological outcomes [8].

Discussion

Research Gaps and Future Directions

The results of this review show that all surveillance guidelines after nephrectomy or ablation due to RCC are still haphazard according to institutions and guidelines. Although follow-up is critical, again, consensus on the widely required schedule or imaging algorithm is not achieved, and under- and over-surveillance frequently occur [1]. It has been indicated that the largest recurrence following ablation is observed in the first year, though most centers do not perform highly effective early imaging that could enhance detection rates [5]. Historic recurrences following nephrectomy emphasize the need for follow-up after five years, but guidelines should recommend follow-up sooner before becoming radar blind on late disease outcomes [2].

The second weakness is the absence of incorporation of new imaging systems into the standardized surveillance structure. Diffusion-weighted MRI and state-of-the-art CT protocols also promise a lower radiation dose without worsening the diagnostic quality, but little is known about their effects on long-term patient outcomes [10]. There is also the possibility of considering the use of biomarkers to customize surveillance, as even in the field of precision oncology, this practice is obtaining increasing attention. In the absence of randomized trials of varying follow-up strategies, clinicians still use institutional or practitioner choices as the basis, which can lead to inefficient care [11].

Patient-centered outcomes are also not well represented. Although the evidence indicating the psychological impact of regular imaging is growing, very few surveillance models include patient-reported outcomes in decision-making. Waiting anxiety and radiation or contrast concern can adversely impact adherence to a follow-up schedule [6]. The solution to these challenges includes stronger qualitative studies and incorporating supportive care into surveillance channels.

Lastly, the lack of emphasis on functional and quality-of-life outcomes is present. The majority of the literature favors oncological outcomes like recurrences and survival, and does not pay attention to the long-term renal functioning or cardiovascular risk in the presence of chronic kidney disease. Surveillance-related protocols that involve tracking of renal activity as well as imaging would offer a more comprehensive picture of patient recovery and patient outcomes [8]. All of these gaps make it clear that the existing data gap demands intensified attention toward multicenter trials, cost-effectiveness studies, and patient-focused research to improve surveillance patterns in renal cancer [13].

Strengths and Limitations of the Study

The present scoping review introduces a cohesive literature review of post-nephrectomy and ablation surveillance protocols to identify variations in the practice and the essential gaps in the evidence. One of its strengths is centered on both surgical and ablative modalities and provides a comparative view of the issue, which is representative of the modern urologic cancerophilic reality [4]. The other strength is that it includes a variety of study designs, such as guidelines, systematic reviews, as well as observational cohorts that enrich a spectrum of analysis.

Nevertheless, restrictions should be accepted. These details (slim evidence base) came down to five studies (finally) used. Such a small pool may limit generalizability and make it difficult to draw adequate conclusions for all clinical situations [15]. Also, the heterogeneity of the included studies (including guidelines for single-center cohorts) interferes with the direct comparison of research. Studies that were not in the English language were not included in the review, potentially missing relevant information from other regions and states, especially Asia, where ablation is becoming prevalent [7].

Absence of full search strings, specific time limits, and a detailed risk-of-bias assessment limits the replicability of the review. Likewise, the methods for data synthesis and study quality evaluation require clearer explanation, and we recognize that the PRISMA flow description needs greater transparency. Lastly, since it was not a meta-analysis, the results reflect a qualitative synthesis of data, but not pooled quantitative results, which can decrease accuracy in measuring the risk of recurrence or investigating survival.

## Conclusions

The use of postoperative surveillance following nephrectomy or ablation for RCC is widely recognized as essential; however, existing guidelines remain inconsistent, fragmented, and often lack strong supporting clinical evidence. Although nephrectomy usually involves a longer-term follow-up (because of the potential possibility of developing late recurrence), ablation has the necessity to reduce to early intensive imaging checks, which would confirm the successful treatment and locate the marginal disease. In both modalities, it is increasingly recognized that a one-size-fits-all model in the future of surveillance is insufficient, and risk-stratified and patient-centered surveillance is well-posed. New imaging modalities are on the rise, and additional research needs to confirm the cost-effectiveness and clinical utility of new modalities in clinical practice. Also, surveillance systems should be adapted to embrace the functional outcomes, patient preferences, and psychological effects, in addition to the oncological control. The low amount of sufficiently eligible studies and the heterogeneous nature of evidence applicability demonstrate the urgent necessity to turn to multicentric collaboration and prospective studies to test and improve follow-up strategies.

In conclusion, the available evidence indicates that although surveillance is essential, its current variability undermines the uniformity of care. Standardizing follow-up protocols for renal cancer, supported by high-quality evidence and guided by patient-centered outcomes, will be critical to optimizing management after nephrectomy or ablation. Until such data are available, clinicians must carefully balance guideline recommendations with individual patient needs, institutional resources, and evolving imaging technologies to provide effective and minimally risky long-term care.
